# Comparative Studies on Polysaccharides, Triterpenoids, and Essential Oil from Fermented Mycelia and Cultivated Sclerotium of a Medicinal and Edible Mushroom, *Poria Cocos*

**DOI:** 10.3390/molecules25061269

**Published:** 2020-03-11

**Authors:** Dongdong Wang, Chonggui Huang, Ye Zhao, Lin Wang, Yongcheng Yang, Anhua Wang, Yang Zhang, Gaosheng Hu, Jingming Jia

**Affiliations:** 1 School of Traditional Chinese Materia Medica, Shenyang Pharmaceutical University, Shenyang 110016, China; wizwddong@mywiz.cn (D.W.); wguton1234@sina.com (Y.Z.); 15241821063@163.com (Y.Y.); sywanganhua@163.com (A.W.); 2 Taizhou Pharmaceutical High-Tech Industrial Park Management Committee, Taizhou 225309, China; huangchonggui@163.com; 3 Heilongjiang Provincial Institute for Food and Drug control, Harbin 150081, China; situhong@hotmail.com; 4 The Sixth Traditional Chinese medicines Factory, Zhongxin Pharmaceutical Group Ltd., Tianjin 300401, China; zhangyang19880702@126.com; 5 Joint Molecular Pharmacognosy laboratory of Shenyang Pharmacetical University and Dong A University, Shenyang 110016, China

**Keywords:** *Poria cocos*, secondary metabolites, content determination, water-soluble polysaccharides, exopolysaccharides, fermented mycelia, triterpenoids, HS-GC/MS

## Abstract

*Poria cocos*, an important medicinal and edible fungus, is well known in East Asia. The main active components are water-soluble polysaccharides (WPS) and triterpenoids. Due to the growing market demand, long cultivation period, and consumption of pine trunk during cultivation, alternative methods for producing *P. cocos* or its active components should be investigated. In this study, WPS, triterpenoids, monosaccharide composition, and essential oil in fermented mycelia and cultivated sclerotium were analyzed using UV spectrophotometry, HPLC, pre-column derivatization, and HS-GC/MS, respectively. Our results showed that the WPS and triterpenoids in mycelia are several times higher than those in sclerotium. Among the 62 compounds identified by HS-GC/MS analysis from the essential oil obtained from the fermentation media and a fresh external layer, the two main fragrances in common were linalool and methyl phenylacetate. Our results suggested that it is applicable to produce polysaccharides and triterpenoids by the fermentation of *P. cocos*, and a strategy to improve triterpenoid production in the fermentation process was proposed.

## 1. Introduction

Medicinal mushrooms are rich sources of therapeutically useful and biologically active agents [1]. For several thousand years, *Poria cocos*, a traditional Chinese medicinal and food mushroom that belongs to the family of Polyporaceae, has been used in China and other East Asian countries for its ability to invigorate the spleen and tonify the stomach; it also has sedative, tranquilizing, and diuretic properties [2]. As reported previously, the chemical constituents of *P. cocos* include polysaccharides [3,4,5,6] and triterpenoids [7,8,9,10]. Basically, there are two kinds of polysaccharides in sclerotium, water-soluble polysaccharides (WPS) (active form, 0.51–2.69%) and alkali-soluble polysaccharides (APS) (inactive form, 32.25–76.64%) [11]. Crude WPS isolated from *P. cocos* was found to reduce the tumor growth by 11.5–18.5% and enhance the antioxidant enzyme activity when fed for 7 weeks at the daily dose of 100–200 mg/Kg in liver cancae male Wistar rats [12]. Recently, APS was reported to improve hyperglycemia, hyperlipidemia, and hepatic steatosis in ob/ob mice via the modulation of gut microbiota [13]. Exopolysaccharides (EPS), another kind of water-soluble polysaccharide, is produced in the media during the liquid fermentation of *P. cocos*, and it has also been reported to have anti-tumor activity both in vivo (Sarcoma-180 solid tumors implanted in BALB/c mice, a immune deficient inbred mice line) and in vitro (HL-60 tumor cells) [6] as well as antioxidant activities [14]. Triterpenoids are reported to be responsible for the diuretic [15], anti-tumor [16], and anti-inflammatory activities [8] of *P. cocos*. More than 50 triterpenoids have been purified from *P. cocos*, derived from lanostane or secolanostane skeletons [17]. A more systematic introduction about the chemistry and pharmacology activity of *P.cocos* has been reviewed [17]. The main constituents are dehydrotumulosic acid (DTA), 3-epi-dehydrotumulosic acid (eDTA), and polyporenic acid C (PAC) [18].

As a consequence of its nutritional and health values, *P. cocos* has gained wide popularity as a nutraceutical and functional food in China. Normally, *P. cocos* grows underground around the roots or dried trunks of pine trees and needs a long cultivation period of about 7 months. In recent decades, the fermentation culture of *P. cocos* has been developed because of the potential for the increased production of mycelia and bioactive components in a compact space and in a shorter period of time. Many studies have been conducted to examine the influence of different conditions on *P. cocos* culture, including medium composition, temperature, and environmental conditions [19,20,21]. The liquid culture of *P. cocos* has been proven as a potential way for the production of triterpenoids [22]. However, there has been no reports simultaneously comparing the terpenoids and polysaccharides as well as essential oil compositions between liquid cultured mycelia and cultivated sclerotium.

In this study, the contents of polysaccharide and triterpenoids and the composition of essential oil were simultaneously compared between fermented mycelia and artificial cultivated sclerotium of P. cocos. Besides determining the polysaccharide content using UV spectrophotometry, we used a pre-column 1-phenyl-3-methyl-5-pyrazolone (PMP) derivatization method to determine the monosaccharide composition of different kinds of polysaccharides from both fermented mycelia and cultivated sclerotium of *P. cocos*. The levels of the three main triterpenoids (DTA, eDTA, and PAC) were determined using reverse phase high performance liquid chromatography (RP-HPLC). In addition, we found that a pleasant fruit-like fragrance was produced during the fermentation process. The volatile compounds were extracted from the fermentation media by water steam distillation and analyzed by GC-MS coupled with a head-space sampler. Then, the main components were identified in the NIST 10 spectral library. The comparative study will provide important and stereo information for the utilization of liquid cultured *P. cocos*.

## 2. Results and Discussion

### 2.1. EPS, WPS, and APS Content Determination

**Figure 1:**
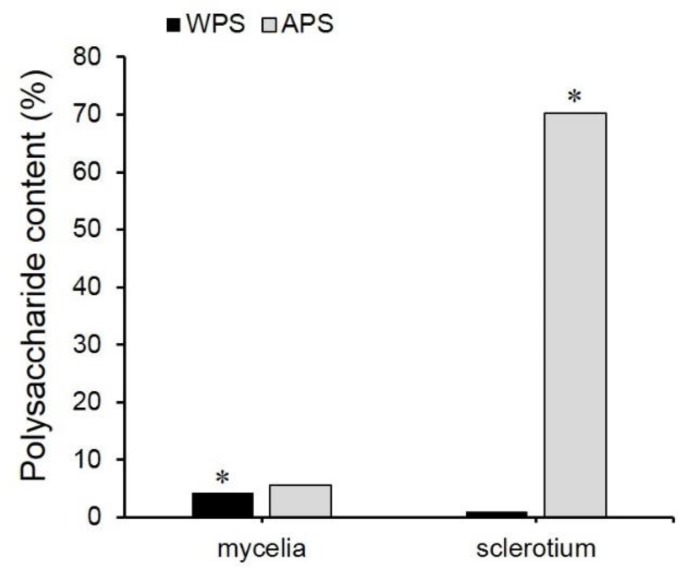
Comparison of the polysaccharide contents (%) in fermented mycelia and cultivated sclerotium of *P. cocos*. Notes: WPS: water-soluble polysaccharides; APS: alkali-soluble polysaccharides; %: g/100 g dry weight. Asterisks indicated the significance (*p* < 0.01).

The WPS and APS contents in cultured mycelia were 4.26% and 5.55% respectively, which were 4.02-folds and 0.08-fold those in cultivated sclerotium (Figure 1). The yield of WPS, APS, and EPS from fermentation was 252.64 mg/L, 329.14 mg/L, and 471.84 mg/L, respectively. As reported previously, WPS is the biologically active polysaccharide in cultivated *P. cocos*. EPS was also reported to exhibit anti-tumor effects. Therefore, the higher content of water-soluble polysaccharides, including WPS and EPS, in fermented mycelia than in cultivated sclerotium would be an important advantage. APS, which is the main component of the cell wall in fungi [23], contributes over 70% of the biomass of cultivated *P. cocos* sclerotium. As reported, the underground conditions, especially environmental pressure, pathogens, and other stress conditions, play important roles in cell wall development and APS accumulation [24].

### 2.2. Monosaccharide Composition Determination

In order to determine the monosaccharide composition, different kinds of polysaccharides (WPS, EPS, and APS) were subjected to pre-column PMP derivatization and analyzed by RP-HPLC. As shown in Table 1, the main components of the water-soluble polysaccharides, including EPS and WPS, from fermented mycelia and sclerotium, are mannose (4.48–27.1%), galactose (5.17–38.48%) and glucose (12.87–89.19%), which is consistent with previous reports [25]. The main component of the APS from the cultivated sclerotium of *P. cocos* is glucose (Table 1). The glucose content reached 97.11%, which is consistent with a previous report that the APS is a β-glucan [26]. However, the glucose content of APS from fermented mycelia of *P. cocos* was much lower (59.8%) and the other major components were mannose (24.8%) and arabinose (11.51%). The results indicated that the mannose and galactose content of both WPS and APS from fermented mycelia are significantly higher than that from sclerotium. It should also be noted that among five kinds of crude polysaccharides, only EPS contained an acidic monosaccharide, glucuronic acid (16.94%). It has been reported that polysaccharides containing acidic monosaccharide showed higher immunology stimulation activity [27]. In the future, the biological activity and the linkage type of different monosaccharides of these polysaccharides should be further studied.

### 2.3. Triterpenoid Content Determination

**Figure 2:**
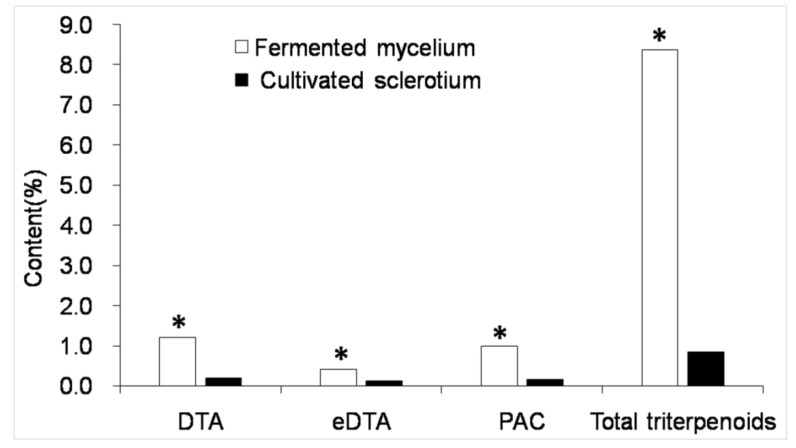
Contents of the three main triterpenoids and total triterpenoids in mycelia and sclerotium of *P. cocos*. Asterisks indicated the significance (*p* < 0.01) compared with cultivated sclerotium.

**Figure 3:**
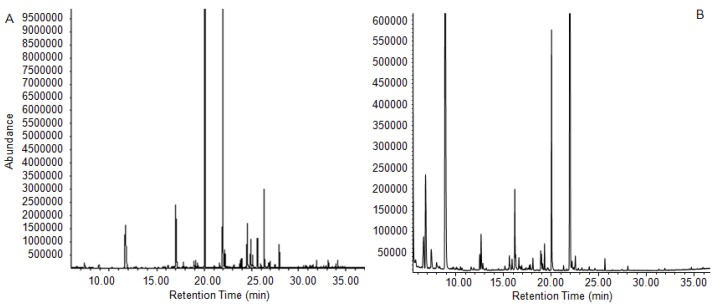
GC/MS chromatogram of essential oil from fermented media (**A**) and a fresh external layer (**B**) of cultivated sclerotium of *P. cocos*.

As demonstrated in Figure 2, DTA (RT = 9.527 min), eDTA (RT = 13.327 min), and PAC (RT = 12.307 min) are separated well by HPLC. Therefore, this method can be used for quantification. There were no apparent differences between the HPLC profiles of cultured mycelia and cultivated sclerotium of *P. cocos*, but the mycelium peak areas were significantly larger under the analytical conditions used here. For cultured mycelia collected on the 17th day after inoculation, the triterpenoid contents were 1.2% (DTA), 0.42% (eDTA), and 1.0% (PAC). This was significantly higher than that in cultivated material: 0.2% (DTA), 0.12 (eDTA), and 0.16% (PAC) (Figure 2). However, according to the UV spectrum of each peak, as shown in Figure 3, peaks 4–8 were also probable triterpenoids. Due to the lack of standard compounds, the peak areas of the unknown compounds (4–8) were recorded and calculated based on the standard formula for DTA. The total triterpenoid content (calculated from peaks 1–8) was 8.36%, which was 9.98 times of that in cultivated *P. cocos* sclerotium (0.84%). The DTA, eDTA, and PAC yields were 71.36 mg/L, 26.41 mg/L, and 60.12 mg/L, respectively, and the yield of total triterpenoids in culture was about 497.14 mg/L. These results demonstrated the advantages for producing useful secondary metabolites using the fermentation of *P. cocos* mycelia. Our previously published result proved that a higher content of triterpenoids in a two-stage cultured mycelia of P. cocos had stronger diuretic activity in rats compared with cultivated sclerotium [28].

### 2.4. Chemical Composition of Essential Oils from the Fermentation Solution

As we know, there have been no reports about the fragrance in *P. cocos* sclerotium, which makes it unexpected when a strong pleasant fruit-like aroma was found during fermentation. When we sampled the cultured mycelia, we found that the aroma was much stronger in the fermentation media than in the mycelia. Therefore, the essential oils were extracted and analyzed with a HS-GC/MS system. The total ion chromatograms are shown in Figure 3. In all, 62 major volatile compounds were identified from the culture media and fresh external layer of *P. cocos* according to the NIST spectral library, including 24 alcohols, 6 aldehydes, 11 esters, 6 alkenes, and 15 other components, as shown in Table 2. The percentage of total identified compounds was 99.96% and 98.03% for essential oils from culture media and a fresh external layer, respectively.

Among the 62 identified components, 25 were considered to be fragrances according to the Dictionary of Flavor and Fragrance [29] shown in bold type in Table 2. These compounds account for 78.76% of the total peak area in culture media essential oil and 54.4% of that in a fresh external layer of cultivated *P. cocos*. The major fragrance components in common were linalool (35.72% in media essential oil and 9.65% in external layer essential oil) and methyl phenylacetate (31.29% in media essential oil and 19.98% in external layer essential oil). Linalool is a naturally occurring monoterpene alcohol found in many flowers and spice plants with wide commercial applications all over the world. Methyl phenylacetate is formed from methanol and phenylacetic acid, and it has a strong odor similar to honey. It occurs naturally in honey, pepper, coffee, and capsicum. In the esters identified from the culture media, methyl jasmonate (0.7%) is also an important signal compound for plants against environmental stress and has been used for the improvement of secondary metabolites in the cell and tissue culture system of many medicinal plants [30]. It has also been reported that the application of methyl jasmonate in the culture media improved the accumulation of triterpenoids in another two important medicinal mushrooms *Inonotus baumii* (Sanghuang in Chinese) [31] and *Ganoderma lucidum* [32] (Lingzhi in Chinese), both belonging to the same family of Polyporaceae with *P. cocos*. Therefore, the reason why the liquid cultured *P. cocos* mycelia secreted this signal compound and the function of this compound in the regulation of secondary metabolites such as triterpenoids needs further investigation.

There are also remarkable differences between essential oils obtained from two origins in which the percentage of 1-butanol, 2-methyl and 1-butanol, and 3-methyl in the essential oil of the fresh external layer of *P. cocos* reached 29.9% and 11.93%, whereas two compounds were not even detected in the media essential oil. A similar situation was found in Bicyclo[3.1.1]hept-3-en-2-one, 4,6,6-trimethyl (1S). Besides, an important fragrance, phenylethanol, was also detected in the fresh external layer rather than the culture media. According to our results, the yield of total essential oil in the fermentation media was 3.6 mL/L. This unexpected result indicated that essential oils, in addition to WPS, EPS, and triterpenoids, are another useful product from the fermentation of *P. cocos*.

**Figure 4:**
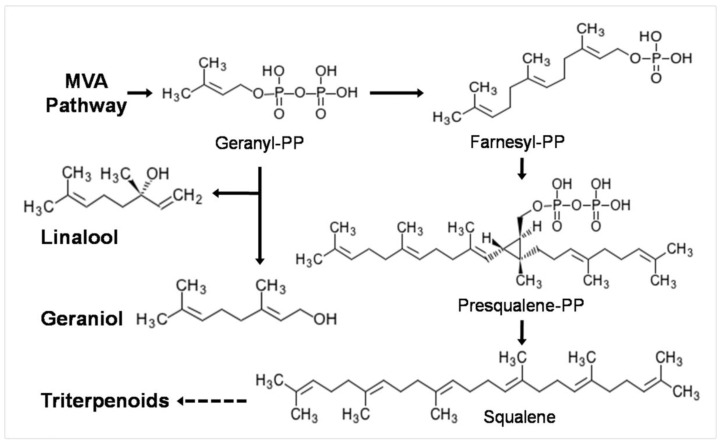
Biosynthetic pathway of main essential oil monoterpene components and triterpenoids in *P. cocos*.

It was reported that in *P. cocos*, terpenes are biosynthesized via the mevalonic acid (MVA) pathway [2]. Monoterpenes and triterpenes are derived from same precursor, geranyl-PP [33], as shown in Figure 4. During the fermentation process, essential oils are secreted into the media and the two main monoterpenes are linalool (35.72%) and geraniol (5.16%). The amount of geranyl-PP required for monoterpene biosynthesis is about 1.17 g/L. However, the total amount of essential oil produced during the whole fermentation process would be even higher than what we measured, because some of the volatile compounds will be lost due to evaporation while shaking the culture. Under the current culture conditions, the total amount of triterpenes produced was 447 mg/L. If the production of essential oil compounds, especially monoterpenes, can be inhibited and the precursors can be converted to triterpenoids instead, then the production of triterpenoids could be much higher. This gives us a reasonable strategy to manipulate the production of triterpenoids through fermentation.

## 3. Materials and Methods

### 3.1. Chemicals and Reagents

Dehydrotumulosic acid (DTA), 3-epi-dehydrotumulosic acid (eDTA), and polyporenic acid C (PAC) were provided by the Kanion Pharmaceutical Co. Ltd. Research Center. Mannose, rhamnose, glucuronide, glucose, galactose, and arabinose were purchased from the National Institute for the Control of Pharmaceutical and Biological Products (Beijing, China).

### 3.2. Fungus Isolation and Culture Conditions

*P. cocos* sclerotium was collected fresh from a cultivation base located in Luotian country, Hubei Province, in September 2013 for fungus strain isolation and October 2018 for comparative content determination, and these were authorized by Prof. Yuan Jiu-zhi in Shenyang Pharmaceutical University. The specimen is stored in the Herbarium of Shenyang Pharmaceutical University, with voucher number SPU-f201309-013 and SPU-f201810-015. Sterilized *P. cocos* sclerotium was inoculated onto potato dextrose agar (PDA) media for 3 days, and growing mycelia were picked up using sterile toothpicks and inoculated onto new PDA plates. Purified *P. cocos* mycelia were maintained on PDA plates. To create a seed culture, mycelia covering an area of 1 cm^2^ were cut from the plates and inoculated into 200 mL of liquid media in Erlenmeyer flasks for 10 days. Then, the seed culture was inoculated into 200 mL of new media in Erlenmeyer flasks at a ratio of 1:50 (*v*/*v*). These flasks were incubated at 25 °C with shaking at 130 rpm for 17 days. The liquid culture medium was potato dextrose broth supplemented with 0.04 g/L vitamin B_1_ and 5 mL/L corn liquor.

### 3.3. Measurements of Biomass and Preparation of EPS, WPS, and APS

Cultured mycelia were filtered through a stainless steel sieve (100 meshes), washed with distilled water three times, then lyophilized, weighed, and recorded as biomass. The first filtrate was collected, and four volumes of 95% ethanol were added. The precipitated polysaccharides were collected by centrifugation at 12,000 rpm for 15 min and then washed sequentially with anhydrous ethanol, acetone, and diethyl ether. This fraction was named EPS. For the preparation of WPS, the dried mycelia or dried cultivated sclerotium were extracted three times with water by sonication for 30 min to obtain the WPS fraction. The debris after WPS extraction was extracted 30 times with 1 M NaOH solution under sonication for 30 min to obtain the APS fraction. The WPS and APS solutions were subsequently adjusted to 80% ethanol, and the precipitates were collected and dried as mentioned above.

### 3.4. Determination of β-d-Glucose Equivalence of EPS, WPS and APS

β-d-glucose was dissolved in distilled water and further diluted to create a series of solutions of known concentration. A490 (X) was recorded after performing the phenol–sulfuric acid assay and was used with the known glucose quantity (μg) (Y) for linear regression analysis to obtain the standard curve. The different polysaccharide fractions from mycelia and cultivated sclerotium were dissolved in distilled water or 1 M NaOH solution and subjected to the phenol–sulfuric acid assay. The A490 was recorded, and the equivalent glucose concentration was calculated from the standard curve. The standard formula was Y = 0.0089X + 0.0224 (R2 = 0.9981), with a linear range of 6.74–80.88 μg. The addition of 1 M NaOH had no significant effect on the results (data not shown).

### 3.5. Monosaccharide Composition Analysis

The monosaccharide composition of the polysaccharide samples was analyzed using 1-phenyl-3-methyl-5-pyrazolone (PMP) pre-column derivatization as described [34]. A sample of polysaccharide (15.0–20.0 mg) was dissolved in 2 M trifluoroacetic acid (2.0 mL) in a 10 mL ampoule. The ampoule was sealed in a nitrogen atmosphere and incubated for 8 h in an oven at 110 °C. Following incubation, the ampoule was cooled to room temperature. Then, the reaction mixture was neutralized to pH 7 with 2 M NaOH. Then, the mixture was brought to 10 mL and filtered through a 0.45 μm Millipore filter. The filtrate was retained for PMP determination.

**Figure 5:**
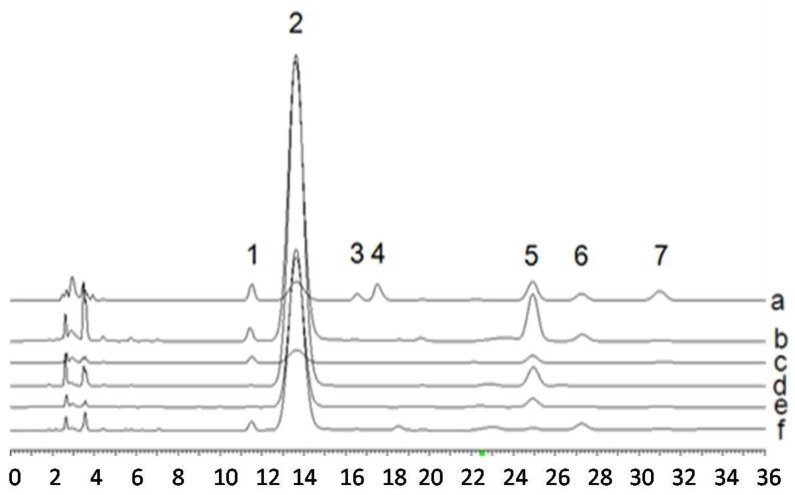
HPLC chromatograms of derivatized products of standard monosaccharide mixtures and the PMP derivatives of hydrolysis products of polysaccharides from *P. cocos* mycelia (M) or sclerotium (S). a: Standard monosaccharide mixture; b: APS (M); c: WPS (M); d: APS (S); e: WPS (S); f: EPS (M). Peak 1: Mannose; 2: PMP; 3: Rhamnose; 4: Glucuronic acid; 5: Glucose; 6: Galactose; 7: Arabinose.

For the PMP reaction, 100 μL of monosaccharide solution or sample solution was mixed with 50 μl 0.3 M NaOH and 50 μL 0.5 M PMP in 1.5 mL tubes and incubated for 40 min at 80 °C in a water bath. The reaction mixture was cooled to room temperature and neutralized with 50 μL 0.3 M HCl. Then, the mixture was extracted three times with 200 μL of chloroform. The aqueous phase was collected and brought to 1 mL with distilled water before filtration through a 0.45 μm Millipore filter. The filtrate was analyzed by RP-HPLC using the following conditions. Mobile phase: ACN/20 mM ammonium acetate solution (Ph = 5.5) (22/78); detection wavelength: 245 nm, flow rate: 1.0 mL/min; column temperature: 30 °C; column type: Dikma Platisil ODS column (250 mm × 4.6 mm, 5 μm). The HPLC chromatogram of monosaccharide composition was shown in Figure 5. The peak area (X) and the corresponding concentration (Y) were subjected to linear regression analysis to give the standard formulas listed in Table 3 together with retention time (RT), linear ranges and correlation coefficient square (R^2^).

### 3.6. Determination of DTC, eDTC and PAC

**Figure 6:**
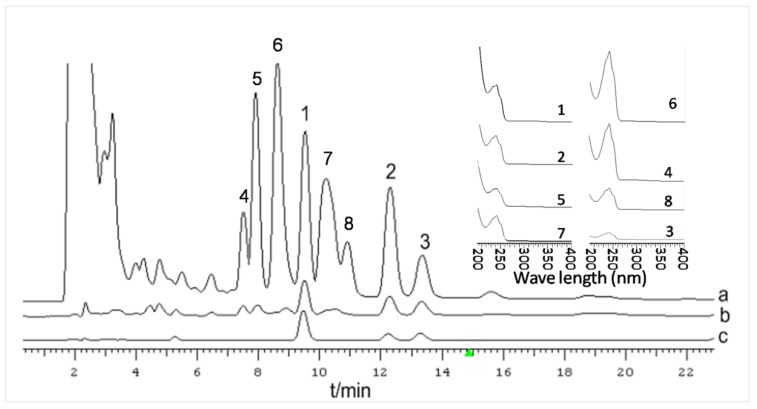
HPLC chromatograms of cultured *P. cocos* mycelia sampled on the 17th day (**a**), cultivated *P. cocos* sclerotium (**b**) and the three standard triterpenoid compounds (**c**). The inset shows the UV spectra of HPLC peaks 1–8. 1, dehydrotumulosic acid (DTA); 2, polyporenic acid C (PAC); 3, 3-epi-dehydrotumulosic acid (eDTA); 4–8 unknown triterpenoids.

A sample (30 mg) of dried mycelium powder or cultivated sclerotium of *P. cocos* was extracted using 1 mL of methanol under sonication for 30 min, followed by centrifugation at 12,000 rpm for 15 min. The supernatant was filtered through a 0.45 μm Millipore filter. Samples were analyzed with Dikma Platisil ODS columns (250 mm × 4.6 mm, 5 μm) at 30 °C with ACN/0.5% phosphate solution (70/30) as the mobile phase. The detection wavelength was 242 nm and the flow rate was 1.0 mL/min. The HPLC chromatogram of the standards used and the sample extract are shown in Figure 6.

To calculate the DTA, eDTA, and PAC content of the *P. cocos* samples, standard curves were prepared using serial dilutions of each of the three compounds. Peak area (X) and compound concentration (Y) were used for the linear regression analysis. The standard formulas of the three standard compounds are listed in Table 4.

### 3.7. HS-GC/MS Analysis of Essential Oil from Fermented Media

Fermentation media (400 mL) was subjected to vacuum evaporation at 45 °C, and the distilled water fraction was extracted with 80 mL of hexane three times. The hexane fraction was combined and dried by the addition of 1 g of anhydrous Na_2_SO_4_; then, it was concentrated at reduced pressure until all the hexane was recycled. The total yield of essential oil was 1.2 mL. For the essential oil from the external layer of fresh *P. cocos*, 300 g of fresh external layer was extracted with distilled water using the distillation method according to the description in the China Pharmacopeia 2015 to obtain 0.28 mL of essential oil. The essential oil obtained was dried by the addition of anhydrous Na_2_SO_4_. Then, 0.2 mL of essential oil was added to a 20-mL vial, which was incubated at 90 °C for 40 min, and then it was analyzed with an Agilent 19091s-433UIHP-5MS capillary column (30 m × 320 μm × 0.25 μm) in an Aglient 7890 GC/5795 MS machine. The injection volume was 1 μL, the split ratio was 5:1, and the gas flow rate was 2.0 mL/min. The temperature of both the injector and detector was 250 °C. The oven temperature was programmed as follows: it was held at 40 °C for 2 min, increased to 260 °C at 5 °C/min, and then held constant at 260 °C for 1 min. Mass spectra were obtained using the electron impact (EI+) mode at 70eV with an ion source temperature of 250 °C. Mass spectra were recorded in the 50–500 range. The structures of the samples were identified by computerized matches in the NIST 10 spectral library. The areas of ion current peaks were determined and normalized into relative peak areas.

### 3.8. Statistical Analysis

All experiments except GC/MS analysis were carried out in triplicate. Statistical analysis was performed with one-way ANOVA to decide a significance of difference among samples at the level of 0.01.

## 4. Conclusions

We compared the levels of major polysaccharides and three triterpenoids in cultured mycelia and cultivated sclerotium of *P. cocos*. We also determined the monosaccharide composition of the different kinds of polysaccharide. According to our results, the contents of WPS and APS in fermented mycelia were 4.02 and 0.08 times that in cultivated sclerotium. Besides, all the crude polysaccharides from mycelia contained a higher ratio of mannose and galactose. Most obviously, only EPS in fermentation media contained glucuronic acid (16.94%) The total triterpenoid content in cultured mycelia was 9.98 times that of cultivated sclerotium. Furthermore, GC/MS analysis results showed that the major fragrance components in common were linalool and methyl phenylacetate. Considering the biosynthetic pathway of terpenes, a possible strategy for improving the yield of triterpenoids in *P. cocos* fermentation was proposed. In summary, fermentation is an attractive alternative method for producing *P. cocos* mycelia and useful secondary metabolites including polysaccharides, triterpenoids, and essential oil. Further activity assay will provide more useful information for their potential application.

## Figures and Tables

**Table 1:** Monosaccharide content (%) of each class of polysaccharide.

	Man	Rha	Glcu	Glc	Gal	Ara
EPS (Me)	27.01	2.07	16.94	12.87	38.48	2.63
APS (My)	24.85	0.32	-	59.81	3.51	11.51
WPS (My)	9.11	1.25	-	67.74	19.81	2.09
APS (S)	2.23	-	-	97.11	-	0.66
WPS (S)	4.48	-	-	89.19	5.17	1.16

-, not detectable; Man, mannose; Rha: rhamnose; Glcu: glucuronic acid; Glc: glucose; Gal: galactose; Ara: arabinose. (My): mycelia; (Me): media; (S): sclerotium.

**Table 2:** Compounds identified in the essential oil extract of culture media and fresh external layer of cultivated sclerotium of *P. cocos*.

Rt (min)	Compound Identified	Formula	Peak Area Percentage (%)	
			Culture Media	Fresh External Layer
Alcohols			47.49	63.18
7.378	1-Butanol	C_4_H_10_O	1.29	-
8.835	**1-Butanol, 3-methyl**	C_5_H_12_O	-	11.93
8.943	1-Butanol, 2-methyl	C_5_H_12_O	1.06	29.90
12.646	1-Pentanol, 4-methyl	C_6_H_14_O	-	0.59
12.659	**1-Hexanol**	C_6_H_14_O	-	0.76
15.706	Glycerin	C_3_H_8_O_3_	-	0.57
15.909	**1-Heptanol**	C_7_H_16_O	-	0.23
16.202	**1-Octen-3-ol**	C_8_H_16_O	0.25	4.74
16.915	Phloroglucitol	C_6_H_12_O_3_	0.27	-
16.921	**3,4-Dimethyl-3-hexanol**	C_8_H_18_O	-	0.31
17.825	**1-Hexanol, 2-ethyl**	C_8_H_18_O	0.61	-
18.098	**Eucalyptol**	C_10_H_18_O	-	0.24
18.919	4-Heptanol, 4-methyl	C_8_H_18_O	0.80	-
19.001	2-Octen-1-ol, (Z)	C_8_H_16_O	0.16	0.27
19.11	1-Octanol	C_8_H_18_O	0.83	-
19.161	Bicyclo[3.1.0]hexan-2-ol, 2-methyl -5-(1-methylethyl)-, (1. alpha., 2. beta., 5. alpha.)	C_10_H_18_O	-	0.69
19.313	**α-terpineol**	C_10_H_18_O	-	1.51
20.064	**Linalool**	C_10_H_18_O	35.72	9.65
20.127	**Phenylethyl Alcohol**	C_8_H_10_O	-	0.88
22.189	**1-Nonanol**	C_9_H_20_O	0.63	-
22.5	**Terpinen-4-ol**	C_10_H_18_O	0.16	0.91
24.53	**Geraniol**	C_10_H_18_O	5.16	-
25.961	2-Pentadecanol	C_15_H_32_O	0.29	-
27.609	3-Tetradecyn-1-ol	C_14_H_26_O	0.23	-
Aldehyde			1.78	2.33
7.423	Heptaldehyde	C_7_H_14_O	-	2.33
11.844	**2-Hexenal, (E)**	C_6_H_10_O	0.47	-
17.704	**Benzeneacetaldehyde**	C_8_H_8_O	0.21	-
21.623	Benzaldehyde, 3-ethyl	C_9_H_10_O	0.57	-
22.093	Isophthalaldehyde	C_8_H_6_O_2_	0.30	-
30.568	5-Methyl-2-phenyl-2-hexenal	C_13_H_16_O	0.22	-
Esters			34.27	21.47
12.512	1-Methoxy-2-propyl acetate	C_6_H_12_O_3_	-	0.86
12.843	**1-Butanol, 3-methyl, acetate**	C_7_H_14_O_2_	0.14	-
16.622	Pentanoic acid, 2-hydroxy-3-methyl-, methyl ester	C_7_H_14_O_3_	0.28	-
19.313	Ethyl 2-(5-methyl-5-vinyltetrahydrofuran-2-yl) propan- 2-yl carbonate	C13H22O4	0.68	-
21.966	**Benzeneacetic acid, methyl ester**	C_9_H_10_O_2_	31.29	19.98
23.951	**Benzeneacetic acid, ethyl ester**	C_10_H_12_O_2_	0.92	-
25.599	Bornyl acetate	C_12_H_20_O_2_	-	0.23
28.01	Isobornyl propionate	C13H22O2	-	0.40
32.667	3,7,11-Trimethyl-3-hydroxy-6,10-dodecadien-1-yl acetate	C_17_H_30_O_3_	0.10	-
34.162	**Methyl jasmonate**	C_13_H_20_O_3_	0.70	-
40.657	**Dibutyl phthalate**	C_16_H_22_O_4_	0.15	-
Alkenes			11.73	0.00
16.756	**beta-Myrcene**	C_10_H_16_	0.16	-
26.394	Cyclooctene, 3-methyl	C_9_H_16_	8.14	-
26.865	5-Undecene, 6-methyl	C_12_H_24_	0.43	-
27.501	Cycloundecene (Z)	C_11_H_20_	0.22	-
27.997	Nonane, 3-methylene	C_10_H_20_	2.20	-
33.17	1-Heptene, 4-methyl	C_8_H_16_	0.57	-
Others			4.73	11.05
12.684	1-Aziridineethanol	C_4_H_9_NO	0.13	-
15.623	**6-methyl- 5-hepten-2-one**	C_8_H_14_O	-	0.40
15.693	N-Methoxy-N-methylacetamide	C_4_H_9_NO_2_	0.35	-
18.919	Vinyl ethyl sulfoxide	C_4_H_8_OS	-	0.32
18.919	Hydrazine, 1,1-dipropyl	C_6_H_16_N_2_	-	1.04
19.307	**Ylangene**	C_15_H_24_	-	0.28
22.138	3-Pyridinecarboxylic acid, 4-hydroxy	C_6_H_5_NO_3_	-	0.18
22.144	1,3-Dimethyl-1-cyclohexene	C_8_H_14_	-	1.05
23.169	**Bicyclo[3.1.1]hept-3-en-2-one, 4,6,6-trimethyl-, (1S)**	C_10_H_14_O	-	7.32
24.641	1,1’-Bicyclopentyl	C_10_H_18_	0.33	-
25.102	Ethanone, 1-(3,4-dimethylphenyl)	C_10_H_12_O	1.35	-
25.611	**2-Undecanone**	C_11_H_22_O	2.07	-
31.255	Phenol, 2,4-bis(1,1-dimethylethyl)	C_14_H_22_O	0.17	-
31.935	Naphthalene, 1,2,3,5,6,8a-hexahydro-4,7-dimethyl-1- (1-methylethyl), (1S-cis)	C_15_H_24_	0.32	-
34.671	p-Hydroxyamphetamine	C_9_H_13_NO	-	0.46

**Table 3:** Regression equations and linear ranges for monosaccharides.

Monosaccharide	RT (min)	Standard Formula	Linear Range (μg)	R^2^
Mannose	11.53	Y = 2E-07X − 0.0053	0.01–0.125	0.9993
Rhamnose	16.56	Y = 3E-07X − 0.0276	0.01–0.125	0.9992
Glucuronide	17.52	Y = 3E-07X − 0.0861	0.0256–0.320	0.999
Glucose	24.93	Y = 3E-07X − 0.0569	0.0532–0.665	0.9991
Galactose	27.27	Y = 2E-07X − 0.0117	0.010–0.13	0.999
Arabinose	30.99	Y = 2E-07X − 0.0054	0.01–0.125	0.9993

**Table 4:** Regression equations and linear ranges of three triterpenoids.

Compound	RT (min)	Standard Formula	Linear Range (μg)	R^2^
DTA	9.527	Y = 1E-06X − 0.0398	0.340–3.40	0.9952
eDTA	13.327	Y = 9E-07X − 0.0022	0.091–0.91	0.9952
PAC	12.307	Y = 1E-06X − 0.0044	0.080–0.80	0.9942
